# Genome Sequence of *Klebsiella oxytoca* SA2, an Endophytic Nitrogen-Fixing Bacterium Isolated from the Pioneer Grass *Psammochloa villosa*

**DOI:** 10.1128/genomeA.00601-13

**Published:** 2013-08-15

**Authors:** Mingyue Chen, Li Lin, Yanming Zhang, Li Sun, Qianli An

**Affiliations:** State Key Laboratory of Rice Biology, Institute of Biotechnology, Zhejiang University, Hangzhou, Chinaa; Guangxi Key Laboratory of Sugarcane Genetic Improvement, Sugarcane Research Institute, Guangxi Academy of Agricultural Sciences, Nanning, Chinab

## Abstract

*Klebsiella oxytoca* strain SA2 is an endophytic nitrogen-fixing bacterium isolated from the pioneer grass *Psammochloa villosa*, which grows in the moving sand dunes of Ordos Plateau, China. The SA2 genome sequence provides the genetic background for understanding its endophytic lifestyle and survival in association with grass in nitrogen-poor environments.

## GENOME ANNOUNCEMENT

*Klebsiella oxytoca* is a known opportunistic pathogen that causes septicemia, pneumonia, and urinary tract infections in humans (1–3). Some *K. oxytoca* strains are known due to their roles in biofuel production (e.g., *K. oxytoca* strain KCTC 1686 [4]) and biological nitrogen fixation (e.g., *K. oxytoca* strain M5a1 [5]).

*K. oxytoca* strain SA2 was isolated from roots of the pioneer grass *Psammochloa villosa*, which grows in the moving sand dunes of Ordos Plateau, China (6). It is able to fix N_2_ in free-living states and in association with plants (7), as well as to colonize tissues of rice, maize, and sugarcane plants (7–9).

The genomic DNA of strain SA2 was extracted and constructed into a 500-bp-insert library, and it was sequenced by using the Illumina HiSeq 2000 sequencing system. Whole-genome sequencing resulted in 45,849,616 high-quality filtered paired-end reads, with an average length of 100 bp and about 800-fold coverage. The filtered reads were assembled *in silico* with the Velvet program (10) and the CLC Genomic Workbench 5.5.1 (CLC bio, Aarhus, Denmark). Gaps between scaffolds and within scaffolds were closed with SSPACE basic 2.0 (11) and GapFiller 1.0 (12). A draft genome containing 72 contigs was obtained based on the strain KCTC 1686 genome (accession no. CP003218) (4).

The draft genome sequence of strain SA2 comprises 5,768,574 bases representing >99.9% of the estimated genome size, and it has a G+C content of 55.9%. A total of 5,449 coding sequences (CDSs) were predicted by using Prodigal 2.60 (13) with the default parameters. Putative CDS functions were identified by using the GenDB annotation engine (14). The chromosome has 3 rRNA operons and 75 tRNAs as predicted with RNAmmer (15) and tRNAscan (16), respectively.

The SA2 genome contains genes for nitrogen fixation, siderophore production, and indoleacetic acid synthesis, which likely function in the survival of the associated bacterium and grass in the nutrition-poor sand dunes. The SA2 genome encodes multiple cell wall-degrading enzymes and multiple enzymes that mediate the elimination of reactive oxygen species generated by the plant hosts, which likely facilitate the bacterial endophytic lifestyle. The SA2 genome encodes components of multiple protein secretion systems but not the type III and type VI secretion systems, which likely cut down the mechanisms for secreting effector proteins and virulence factors into plant and animal hosts. Together, the genome sequence of strain SA2 provides the genetic background for understanding the association between the endophytic bacterium and the pioneer grass in nitrogen-poor environments and for future application of the beneficial bacterium to enhance crop nutrition and production.

### Nucleotide sequence accession numbers.

This whole-genome shotgun project has been deposited at DDBJ/EMBL/GenBank under the accession no. ATNG00000000. The version described in this paper is the first version, ATNG01000000.
